# Prevalence of Oral Lesions and Correlation with Intestinal Symptoms of Inflammatory Bowel Disease: A Systematic Review

**DOI:** 10.3390/diagnostics9030077

**Published:** 2019-07-15

**Authors:** Dorina Lauritano, Elisa Boccalari, Dario Di Stasio, Fedora Della Vella, Francesco Carinci, Alberta Lucchese, Massimo Petruzzi

**Affiliations:** 1 Department of Medicine and Surgery, Centre of Neuroscience of Milan, University of Milano-Bicocca, 20126 Milan, Italy; 2 Multidisciplinary Department of Medical and Dental Specialties, University of Campania-Luigi Vanvitelli, 80138 Naples, Italy; 3 Interdisciplinary Department of Medicine, University of Bari, 70121 Bari, Italy; 4 Department of Morphology, Surgery and Experimental Medicine, University of Ferrara, 44121 Ferrara, Italy

**Keywords:** inflammatory bowel disease, Crohn’s disease, ulcerative colitis, oral manifestations

## Abstract

Background: Extra-intestinal manifestations of inflammatory bowel disease (IBD) are widely studied. Oral manifestations are manifold, miscellaneous, and hardly detected by general practitioners and gastroenterologists. Objectives: The main purpose of this systematic review is to find all the possible correlations between inflammatory bowel disease and the oral cavity in order to underline the importance of multidisciplinary cooperation with dental care providers, and to secure better treatments for patients. Materials and methods: Articles were searched up to June 2019 through Ebsco’s, Google Scholar, and PubMed databases. The search terms included IBD, oral manifestations of inflammatory bowel disease, oral manifestations of Crohn’s disease or Ulcerative colitis, an extra-intestinal manifestation of IBD, oral Crohn’s disease, and paediatric inflammatory bowel disease. Discussion: The prevalence of the oral manifestation of IBD ranges from 0.7% to 37% in adults and from about 7% to 23% in children. They can be divided into specific manifestations (cobblestoning mucosa, mucosal tags, cheilitis granulomatosa, pyostomatitis vegetans) and nonspecific manifestations (halitosis, dysphagia, aphthous ulcerations, deep oral fissuring, cheilitis angularis, taste changes, lichen planus). Moreover, the link between IBD and the higher prevalence of dental caries and periodontitis have also been studied. Conclusions: The presence of oral manifestations that precede or follow intestinal symptoms of IBD, must be taken into serious consideration from both gastroenterologists and dentists in order to allow for early diagnosis and improve patients’ quality of life.

## 1. Introduction

Inflammatory bowel disease (IBD) is a group of nosological entities that comprises two major pathological conditions affecting the gastrointestinal tract (GI): Crohn’s disease (CD) and Ulcerative Colitis (UC). Its etiopathological mechanism is still unclear, but it is thought to be multifactorial, involving genetic, environmental, bacterial and immune response connections to the microbiota.

It can affect the entire GI tract, from mouth to anus, primarily involving the small intestine (CD) or the large intestine (UC). The age of onset of symptoms usually varies between 15 and 30 years [1], but the disease can occur at any age.

The main symptoms are chronic diarrhoea (often bloody), abdominal pain, weight loss, fever, secondary anaemia, and fistulas. It can also have extra-intestinal manifestations (EIMs), with a prevalence rate varying widely from 16.7% to 40% [2,3], affecting the eyes, skin, joints, and the oral cavity and it appears to be more common at the onset in paediatric patients [2]. The different ages of study participants and the skills of the examiners performing the evaluation are the causes of that wide variability in assessing it’s prevalence.

Oral manifestations of IBD can be specific or nonspecific, due to intestinal malabsorption or induced by pharmacological treatments.

A few of these manifestations, such as aphthae, buccal mucosal swelling, mucosal tags, deep liner ulcerations, and cobblestoned oral mucosa are more indicative of CD, while pyostomatitis vegetans is correlated to UC.

Especially, orofacial granulomatosis (OFG), a rare condition characterised by swelling of the lip and the oral cavity, must be investigated in young children, because it can conceal underlying Crohn’s disease or be a presenting feature of other systemic diseases [4].

Oral manifestations might precede IBD’s diagnosis (12.7–21%) [2,5], or coincide with it and interfere with therapy.

A proper oral evaluation in paediatric patients must be conducted, particularly in children with OFG, in order to ensure early diagnosis and appropriate treatments.

Moreover, several studies have also demonstrated a higher frequency of dental caries and periodontal disease [6,7,8,9].

Objective: The aim of this review is to highlight all the various aspects and possible links between IBD and oral manifestations, in order to evaluate the role of dentists and dental cares in improving patients’ oral health and consequently their quality of life.

All the various aspects and possible links between IBD and oral manifestations were investigated in a number of case-control, cohort, and cross-sectional studies.

## 2. Materials and Methods

A systematic review was conducted up to June 2019, according to the Preferred Reporting Items for Systematic Reviews and Meta-Analyses (PRISMA) statement guidelines [10], using the databases EBSCO-host, PubMed, and Google Scholar. The search terms included IBD, oral manifestations of inflammatory bowel disease, oral manifestations of Crohn’s disease or Ulcerative colitis, an extra-intestinal manifestation of IBD, oral Crohn’s disease, and paediatric inflammatory bowel disease.

Studies included in this review matched the predefined criteria according to the PICOS (patients, intervention, comparator, outcomes, study design) process as reported in Table 1.

The exclusion criteria were review papers, case reports, papers published before 2000, papers not written in English, papers not mentioning oral manifestations, papers including other bowel diseases not universally recognised among IBD, such as Behçet syndrome. Papers linking the connections between disorders of the oral cavity and IBD in patients of all ages were included in this review.

From each study, data about the demographics of participants (age and sex), oral sign and symptoms, caries and periodontal indices if detected, pharmacological treatments prior to the study, and that could have influenced the symptoms and smoke habits were collected.

A study was found eligible when it fit all the inclusion, and none of the exclusion criteria. Duplicates were removed and they were screened by the title, abstract, and full text. Once a paper was found to fit the eligibility criteria, its references were, in turn, screened in order to find new papers previously missed.

**Figure 1:**
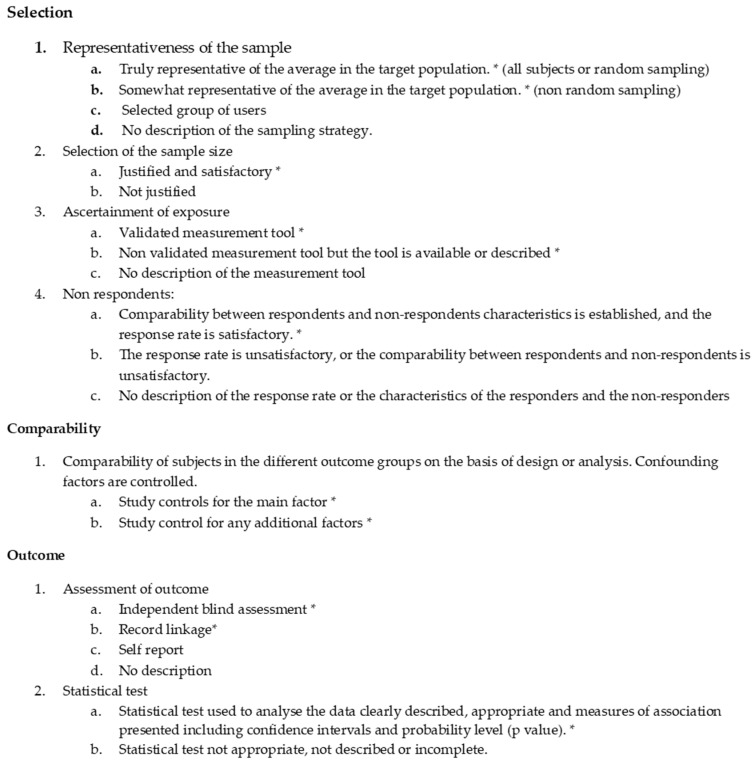
Newcastle Ottawa scale modified for cross sectional studies.

The risk of bias in each individual study was assessed using the Newcastle Ottawa scale (NOS) [11]. The quality score for every single study is reported in Table 2 and Table 3 and in a customized modified version for cross sectional studies (Table 4) as explained in Figure 1. A low score does not necessarily denote an inappreciable study since it is the result of very strict and structured guidelines.

## 3. Results

**Figure 2:**
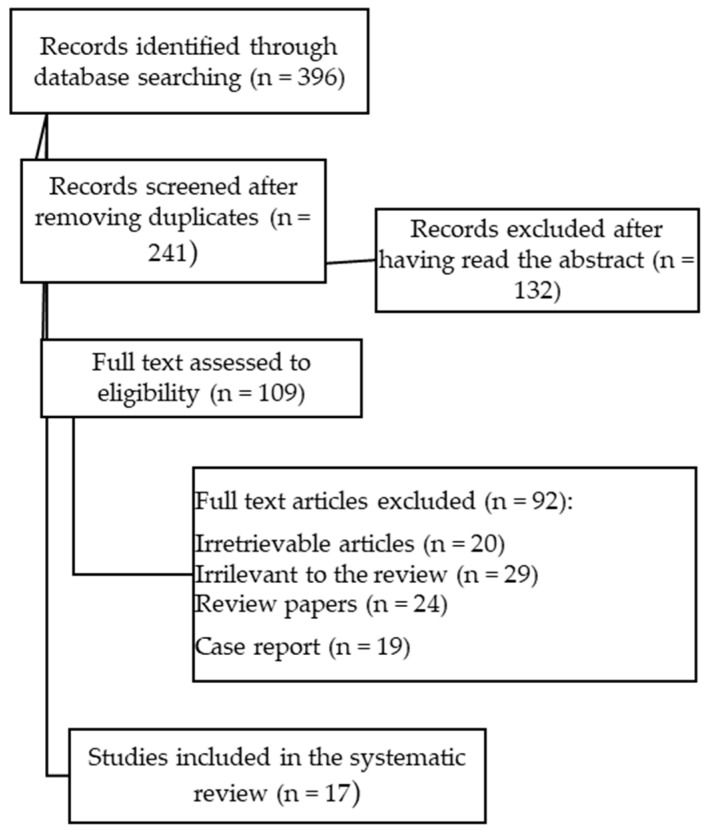
PRISMA flow diagram.

In this review, seventeen papers following the search criteria were included, and data collected in 15 different countries, up to a total of 6692 study participants (2174 of which were under 18 years old). The database research identified 396 studies, but as depicted in the flow diagram (Figure 2), after removing the duplicates and screening the abstracts, only 109 were fully read and 92 excluded because they were irretrievable, irrelevant, or were not eligible after applying the exclusion criteria. Among the 17 papers, there were one cross-sectional study, one prospective study, four retrospective studies, eleven case-control studies with a total of 5369 (3558 with CD and 1811 with UC/indeterminate colitis) patients with IBD among all the different studies.

In all the seventeen studies selected for the review, the main purpose was to identify oral signs and symptoms related to IBD (whether they were early manifestations of a systemic disease or they coexist with the main medical condition) and the secondary purpose was to relate IBD to two main oral conditions, caries, and periodontitis.

As the study design, participants, primary, and secondary outcome varied markedly and the results were poorly distributed, we preferred not to perform a meta-analysis of the studies and rather focused on a description of their results, their limitations, and the possible developments.

Each outcome for every single parameter considered in the systematic analysis, as already described, is observed in Table 5.

## 4. Discussion

The prevalence of oral manifestations in IBD has been reported to range widely from 0.7% to 37% [3,19] in adults and from 7.3% to 23% in children [2,9], data increasing up to 41% when considering only CD children [12].

The remarkable variability in the prevalence reported among the various studies may be due to the different design of the studies, the number and type of population, and in particular, the lack of experience of certain physicians (often gastroenterologists and not dentists) in finding and accurately classifying oral manifestations [12], and in the lack of uniqueness in classifying IBD oral changes, using different parameters, and therefore, making it difficult to compare the different results.

Oral manifestation of IBD can be specific or nonspecific (Table 6) and can precede (12.7–21%) [2,5], or be the presenting sign of gut involvement. Mucocutaneous findings, mostly oral manifestations, can be asymptomatic presenting signs of IBD.

**Figure 3:**
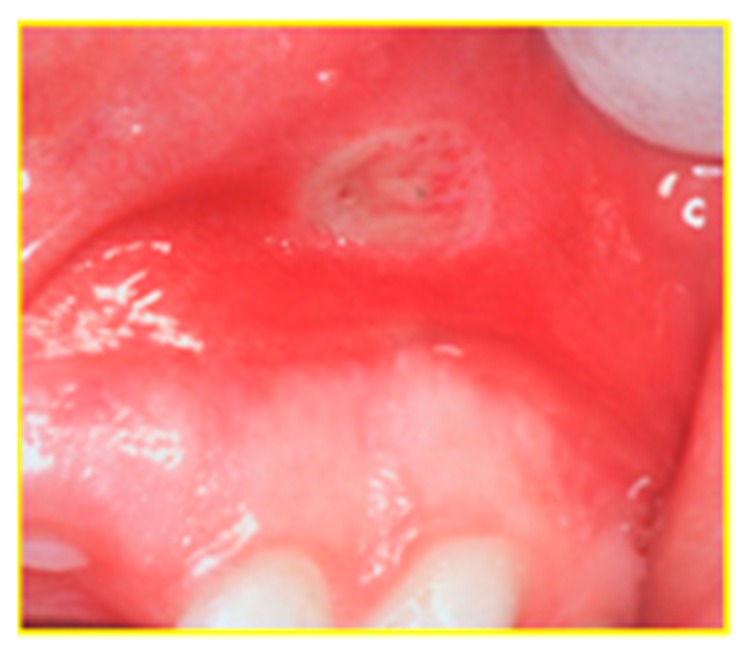
Oral manifestations of IBD: apthous stomatis in the vestibular sulci.

The most common oral manifestation of IBD is aphthous stomatitis (aphthae with an atypical presentation or clinical course) [20] which occurs from about 0.7% to 20% [2,13] (this last one regarding only UC) in adults and from 3.2% to 41.7% in children [5,12]. They cannot be differentiated from regular aphthae unless a biopsy is performed, which must be based on clinical grounds, and thereby, is not always conducted if a histological IBD diagnosis is already available; they are as frequent as in the healthy population, but they have a tendency of recurring in time [19] and resemble the ulcers present in the gastrointestinal tract. They are described as the presence of small, painful but benign oral ulcers, circumscribed by an erythematous halo, and they can be isolated or part of recurrent aphthous stomatitis [20]; they usually occur on the buccal and labial mucosa or the vestibular sulci (Figure 3). In IBD patients, aphthous ulcerations may be caused by a deficit of iron, zinc, or vitamin B12 due to intestinal malabsorption and rectal bleeding linked to the main disease, or a side-effect of their pharmacological treatment. In children, the presence of oral ulcerations is not only painful, but has a significant impact on nutrition and can result in growth failure, development problems, and psychological adjustment.

Furthermore, in children, aphthous stomatitis has proven to be the second most common EIM's [2,5] with a range from 3.2% to 7.3% of all patients with IBD, and the second most common EIM's to appear before diagnosis (21% according to Jose et al. [5] and 29% of cases for Greuter et al., [2] after peripheral arthritis.

The debate is still open as to whether there is a link between the presence of aphthous ulcers and the activity phase of diseases (measured using the Harvey Bradshaw index, the Crohn's disease activity index for CD, the Montreal index of activity, or the Truelove and Witts criteria for UC). According to Laranjera et al. [15], aphthous ulcerations are the most common oral lesions in the active phase of the disease (*p* = 0.001) and oral symptoms were reported in 70.6% of patients in the active phase also, compared to 52.1% in remission with a particular association with dysphagia, regurgitation, and acidic taste. Elahi et al. [13] reported a higher statistical significance (*p* = 0.001) with oral ulceration in patients with severe UC compared to the controls. These data don't find validation in studies by Zervou et al. [18] in which, although a correlation between IBD and oral manifestations was found, it wasn't related to the active or inactive disease. Similarly, Grössner-Schreiber et al. [7] reported that only 38% of their study population showed a link between any oral lesion and IBD activity disease. Brito et al. [6] didn't observe any difference in oral lesions between IBD's and control groups and no correlations with disease activity.

Therefore, any patient presenting a history of recurrent oral ulceration or referring any other GI symptomatology should be sent to a specialist evaluation. Topical applications of corticosteroids seem to give benefits along with systemic treatment of main bowel disease.

Among the specific lesions correlated to CD, there are mucosal tags, cobblestoning of the mucosa, and buccal swelling linked to granulomatous cheilitis. Pyostomatitis vegetans is, on the contrary, mostly noted in UC patients.

Cobblestoning of the mucosa is defined by a combination of deep, transversely, and longitudinally oriented ulcerations separating intact portions of mucosa giving the resemblance of cobblestones [21], and it can be seen in a range from 6% to 20%, respectively in children and adults [12,18].

Pyostomatitis vegetans is a rare oral disorder firstly observed by Hallopeau in 1898 and characterised by the presence of multiple white or yellow pustules on erythematous mucosa that undergo necrosis, rupture, and thus, result in a snail-track appearance [16].

It affects mostly UC patients and it appears on the labial and buccal mucosa, soft and hard palate, gums and rarely on the floor of the mouth. Histologically, it shows acanthosis, intraepithelial, and subepithelial abscesses with neutrophils and eosinophilic infiltrate [16]. As many of the oral manifestations of IBD, it resolves itself with adequate control of the underlying bowel disease.

Another distinctive feature that can appear years before a proper diagnose of CD is cheilitis granulomatosa present in orofacial granulomatosis (OFG). Firstly identified by Wisenfeld et al. in 1985 [22], OFG is a complex condition characterised by a non-caseating granulomatous inflammation, detected by a biopsy and affecting the oral cavity, particularly the lips, with no evidence of systemic involvement. It encompasses other diseases such as granulomatous cheilitis (GC) of Meischer, Melkersson-Rosenthal syndrome (MRS), oral Crohn’s disease, sarcoidosis, tuberculosis, and other infections and allergic conditions.

The main symptoms are recurrent and persistent buccal swelling (mainly lip swelling with angular cheilitis), gingival enlargements, oral ulcers, and sometimes facial palsy and cervical lymphadenopathy. Notably in young children, the occurrence of OFG, after having dismissed other possible infectious or allergic aetiology, is likely linked to Crohn’s disease—in a report of six patients diagnosed with OFG, four developed CD within a few months [4]. In view of this, some authors suggest that all patients with OFG undergo a biochemical test (in particular faecal calprotectin test) and eventually a gastrointestinal endoscopy if symptoms are equivocal for a differential diagnosis between an early oral presentation of Crohn and OFG [4]. The treatments are not specific and include corticosteroids, topical with intralesional injections or systemic, thiopurines, and anti-Tumor necrosis factor-α (TNF-α) therapies. Sometimes, in the severe case of OFG, cheiloplasty may be taken into account.

**Figure 4:**
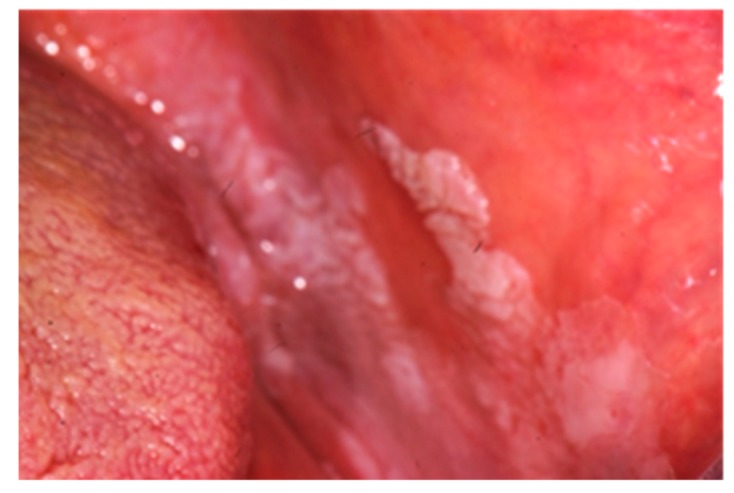
Oral manifestation of IBD: lichen planus in the left buccal mucosa.

Oral lichen planus is a chronic inflammatory dermatosis that affects the oral mucosa and has six different clinical presentations, it can be reticular, erosive/ulcerative, bullous, plaque-like, papular, or atrophic (Figure 4). The aetiology is still unknown, but it is thought to being related to an immune-mediated mechanism that involves dendritic cells and T cells. Oral lichenoid drug reactions have proven to be related to mesalazine and sulfasalazine [16] used in the therapy of IBD patients.

In addition to oral lesions, there is a large group of oral symptoms, such as acidic taste, taste changes, halitosis, dry mouth, and xerostomia. An Iranian study from Elahi et al. [13] showed a significant statistical relationship particularly among halitosis (*p* = 0.001) and taste changes (*p* = 0.001) in UC adult patients with a statistical trend regarding the other parameters. Data were confirmed by Laranjera et al. (16) in an IBD sample with a prevalence of 54.9% in IBD patients and 29.3% in the control group (*p* = 0.011) and Katz et al. [14]. In this last study, beyond the statistical significance of halitosis in UC patients, nausea was identified in CD and vomiting, regurgitation, dry mouth, dysphagia, and geographic tongue were reported to have a statistical trend. Changes in the tongue (a hypotrophic or coated tongue, angiomas, fissures) have also been observed and are considered side effects of the iron and vitamin deficiency caused by rectal bleeding. Taste changes may be sometimes attributed to Metronidazole, an antibiotic used in the therapy of Crohn’s disease. These data must be taken cautiously because they are all based on questionnaires filled out by the patients, and consequently, there might be a bias in their ability to distinguish and report oral symptoms carefully.

In regard to dental caries, a large, prospective Greek study from Koutsochristou et al. [9] analysed decayed, missing, filled teeth indexes for the permanent dentition (DMF-T) and decayed, missing, filled teeth indexes for the the primary dentition, (dmf-t) and found higher values of DMF-T and dmf-t in children and adolescents with IBD (*p* < 0.001); nevertheless, the PCR index researching the presence of plaque was not statistically different from controls. These data find validation in another report from Brito et al. [6] on adult patients (*p* < 0.0001 for CD patients and *p* = 0.003 for UC patients). Grössner-Schreiber et al. [7]; however, using the (decayed, missing, filled surface (DMF-S) a modified version of the DMF-T index which is calculated per tooth surface) showed no difference in DMF-S index between IBD’s and controls even though the first ones had higher plaque index and higher prevalence of dentine caries (46% in IBD vs. 22% in controls).

Furthermore, Szymanska et al. [8] studied two groups of CD patients who either had or had not undergone resective surgery and compared them to healthy controls. CD patients who had undergone resective surgery had higher levels of DMF-S compared to controls and higher levels of plaque (reported using the visible plaque index), but in patients who had not undergone resective surgery, that was not true. The author correlated that outcome to the severity of the disease (in fact, there was a significant, although the weak correlation between the disease duration and the DMF-S index) and the presence of old restorations, a risk factor for new caries.

The increase in dental caries risk is thought to be associated to dietary habits, changes in salivary and microbiological conditions of the oral cavity and malabsorption. Malabsorption of vitamin D, which has been proved to play a significant role in IBD patients [23], may possibly be related to the complex multifactorial etiopathology of dental caries [24], but more studies are required to assess the causative relationship between them. Furthermore, in order to mitigate the intestinal symptoms, patients usually switched to a fat-reduced and richer, refined carbohydrate diet, thus, increasing the risk of caries [17].

Mucogingivitis, an inflammation of the gingival tissues with an oedematous, erythematous, granular, and hyperplastic gingiva was observed by Harty et al. [12] in 12 of 48 children with CD and it’s one of the most common features of oral IBD. Zervou et al. [18] found a higher incidence of gingivitis and gingival bleeding in CD, but not in UC.

A positive association between periodontitis and IBD has also been investigated. Habashneh et al. [1], in their sample population of Jordanian adults, observed that periodontitis was more severe and generalised, especially in UC patients. In particular, the average gingival recession was the most significant data and was higher in UC patients compared to those with CD (*p* = 0.007) and to controls (*p* < 0.005), the average gingival index and plaque index were also higher than the control population. These results have been mirrored by another study from Brito et al. [6] who focused on the effect of smoking as a modifier. Overall periodontitis was more common in IBD patients than controls, but it reached significance among smoking patients with UC versus smoking controls (95.2% versus 70.6%, *p* = 0.008), whilst this difference was not statistical in non-smoking UC and CD patients and controls (*p* = 0.057). Probing pocket depth (PPD), even after being adjusted for race, smoke, gender, age, and plaque, was still higher in CD and UC patients (*p* < 0.0001). These results; however, collided with a previous study from Grössner-Schreiber et al. [7] which revealed similar PPD in IBD patients and a control population that reached no significance and similarly other parameters, such as sites with CAL (clinical attachment loss) > 4, were higher in IBD but did not reach significance.

Koutsochristou et al. [9] by contrast detected periodontitis in a population of children and adolescents in remission with the CPITN index (community periodontal index of treatment needs, an epidemiological index) in a population of children and adolescents, and found that none of them had a healthy periodontium (CPITN score 0), 36% had a CPITN score 1 (meaning only gingival bleeding), 54% had CPITN score 2 (gingivitis with gingival bleeding, calculus, and overhanging restorations), and 9% had CPITN score 3 (at least 1 site with PPD values between 4 and 5 mm). Whereas 40% of the control population had a healthy periodontium, 14% had gingivitis, and none of the subjects had 1 site with pocket. These data showed high statistical significance in patients compared to controls (*p* < 0.001).

A correlation between IBD and periodontitis has, therefore, been determined, even though findings are not uniform, but in order to realise whether there are clinical implications for the management of periodontitis in patients with IBD further studies, on a larger scale, are necessary.

Moreover, there are a few case reports indicating a correlation between TNF-α inhibitors and oral lichen planus [25] and between Infliximab (another TNF-α inhibitor) and oral ulcerations since it has been demonstrated to be immunogenic and leads to antibodies to infliximab related to painful oral ulcerations [26]. Although case reports have been excluded in this review, we think that these two studies may be worthy for further consideration, since no observational studies have been published.

Furthermore, links between the oral cavity and IBD aren’t restricted to mucosal involvements but affect the composition of the oral microbiome. A study from Docktor et al. [27] in a cohort of 114 children demonstrated that CD patients, as opposed to UC and healthy patients, experiment a reduction of the biodiversity of the oral microbiome and particularly some phyla (Fusobacteria and Firmicutes) as happens in the intestinal microbiome. In regard to the oral manifestation, CD patients also have higher levels of anti-Saccharomyces cerevisiae antibody (ASCA), and this is also associated with an increase in disease activity. Russel et al. [28] examined 301 Scottish children and detected a correlation between a positive ASCA status and a severe grade of the disease. ASCA status may then be considered a useful marker for stratifying disease and potential treatments.

## 5. Conclusions

The presence of oral manifestations and symptoms in IBD, along with the higher risk of caries and periodontitis, must be taken into serious consideration. The cooperation of both gastroenterologists and dentists is crucial in order to achieve early diagnosis, better management of therapies, and improve patients’ quality of life. Further developments in research may also involve the search for new therapies and their possible impact on oral health.

## Figures and Tables

**Table 1:** PICOS = patients, intervention, comparator, outcomes, study design.

Parameter	Inclusion Criteria	Exclusion Criteria
Patients	Patients from 0 to 99 years with IBD	Patients with another bowel disease not universally recognized as IBD
Intervention	Not applicable	
Comparator	Not applicable	
Outcomes	Diagnostic accuracy Connections between oral pathology and IBD Relate IBD to caries and periodontitis	
Study design	Prospective, retrospective or concurrent cohort studies Cross sectional studies	Reviews, expert opinion, comments, letter to editor, case reports, conference report. Studies not published in English Studies published before 2000

**Table 2:** Assessment of the quality in cohort studies. Each item is given one star, except comparability which is given a maximum of 2 stars, up to nine total stars. In order to have an outcome all the patients must be exposed to inflammatory bowel disease (IBD), so the item regarding the selection of non exposed cohort is zero for all the studies taken into consideration.

Study	Selection				Comparability	Outcome			Total Score
	Representativeness of Exposed Cohort	Selection of Non-Exposed Cohort	Ascertainment of Exposure	Demonstration that Outcome of Interest was not Present at Start of Study	Comparability of Cohort on the Basis of the Design or Analysis	Assessment of Outcome	Was Follow-Up Long Enough for Outcomes to Occur	Adequacy of Follow-Up of Cohorts	
Greuter et al. [2]	*	/	*	/	* (age)	*	*	*	6/9
Harty et al. [12]	*	/	*	/	** (age, sex)	*	/	/	5/9
Jose et al. [5]	*	/	*	/	** (age at diagnosis, type of EIM)	*	*	/	6/9
Khouri et al. [4]	/	/	*	/	/	*	*	/	3/9
Zippi et al. [3]	*	/	*	/	** (sex, age at diagnosis, clinical history, smoking habit, EIM)	*	*	*	7/9

Legend: * = presence of the outcome / = absence of the outcome EIM= extra intestinal manifestation.

**Table 3:** Assessment of the quality in case-control studies. Each item is given one star up to a total of nine stars.

Study	Selection				Comparability		Exposure			Total Score
	Case Definition Adequate	Representativeness of the Case	Selection of Controls	Definition of Controls	Main Factor	Additional Factor	Ascertainment of Exposure	Same Method of Ascertainment for Cases and Reports	Non Response Date	
Brito et al. [6]	*	*	/	*	*(age)	(other factors)	*	*	/	7/9
Elahi et al. [13]	*	*	/	*	*(age)	*(sex)	*	*	/	7/9
Grössner-Schreiber et al. [7]	*	*	/	*	*(age)	*(other factors)	*	*	/	7/9
Habashneh et al. [1]	*	*	/	*	*(age)	*(other factors)	*	*	/	7/9
Katz et al. [14]	*	/	/	*	*(age)	*(sex)	*	*	/	6/9
Koutsochristou et al. [9]	*	/	/	*	*(age)	*(other factors)	*	*	/	6/9
Laranjeira et al. [15]	*	*	/	*	*(age)	*(other factors)	*	*	/	7/9
Mohan Kumar et al. [16]	*	/	/	*	*(age)	*(sex)	*	*	/	6/9
Rikardsson et al. [17]	*	*	/	*	*(age)	*(sex)	/	*	*	7/9
Szymanska et al. [8]	*	*	/	*	*(age)	*(other factors)	*	*	*	8/9
Zervou et al. [18]	*	/	/	*	*(age)	*(sex)	*	*	/	6/9

Legend: * = presence of the outcome / = absence of the outcome.

**Table 4:** Assessment of quality in cross sectional studies. Each item is given one star, except comparability which is given a maximum of two stars, up to a total of nine stars.

Study	Selection				Comparability	Outcome		Total Score
	Representativeness of the Sample	Sample Size	Ascertainment of Exposure	Non Respondents	Comparability of Subjects on the Basis of the Design or Analysis	Assessment of Outcome	Statistical Test	
Oviedo et al. [19]	*	*	*	/	** (age, sex)	*	/	6/9

Legend: * = presence of the outcome / = absence of the outcome.

**Table 5:** List of studies analysed in this review.

	Study	Design; Setting	Patients *n* (M/F); Mean Age	IBD Patients *n* (M/F); Mean Age	Oral Sign and Symptoms in IBD Patients	DMFTe dmft Index	Periodontal Manifestations	Pharmacological Treatments	Smoke Habits
**1**	Brito et al. [6]	CC; Brazil	253 (88/165); 40.8 ± 22.5 yr Cr = 74 (24/50); 40.3 ± 12.9 yr	CD=99 (31/68); 39 ± 12.9 yr UC = 80 (33/47); 43.3 ± 13.2 yr	Candidiasis = 20 (8 CD, 8 UC, 4 Cr) NS ulcerous aphtous = 3 (2 CD, 1 UC) NS lichen planus = 5 (1 CD, 3 UC, 1 Cr) NS	CD = 15.1 ± 7.2 (*p* = 0.018) UC = 16.4 ± 6.6 (*p* < 0.0001) Cr = 12.5 ± 6.8	CD :PPD = 2.3 ± 1.3 mm (*p* < 0.0001) CAL = 0.9 ± 0.9 mm NS % of sites with BOP = 19.6 ± 20.5 (*p* = 0.038) Periodontitis = 81 (81.8%) Cr: PPD = 1.6 ± 0.4 CAL = 1.2 ± 1.0; % of sites with BOP = 24.4 ± 29.7 Cr periodontitis = 50 (67.6%). UC :PPD = 2.3 ± 0.4 (*p* < 0.0001) CAL = 1.3 ± 1.4 (*p* = 0.004) % of sites with BOP = 21.5 ± 21.9 NS periodontitis = 72 (90%) (*p*<0.001)	Aminosalicylates, immunomodulators, corticosteroids, antibiotics, anti TNF alpha	CD: smokers = 12 (12.1%) non-smokers = 63 (63.3%) former smokers = 24 (24.3%). UC: smokers = 7 (8.7%) non-smokers = 38 (47.5%) former smokers = 35 (43.8%) Cr: smokers = 9 (12.2%) non-smokers=57 (77%) former smokers = 8 (10.8%)
**2**	Elahi et al. [13]	CC; Iran	100 (54/46); 39 ± 25,6 yr. Cr = 50 (26/24); 40 ± 20 yr	UC = 50 (28/22); 38 ± 16 yr	Oral ullerations = 20 (*p* = 0.028); tongue coating = 14 (*p* = 0.012); dry mouth = 30 (*p* = 0.023); halitosis = 34 (*p* = 0.001); acidic taste = 20 (*p* = 0.008), taste changes = 20 (*p* 0.001)	NN	NN	NO	NN
**3**	Greuter et al. [2]	R; Switzerland	329 (181/148); 12 yr 55 had at least 1 EIMs (39 CD, 12 UC, 4 NS)	CD = 173 (104/69); 12 yr. UC/ND = 156 (77/79); 11 yr	aphtous stomatitis = 24 (5 CD, 18 UC,1 NS) (7.3% of the entire study population but 43.6% of 55 patients with EIMs	NN	NN	5-ASA, antibiotics, steroids, immunomodulators, anti-TNF	NN
**4**	Grössner-Schreiber Et al. [7]	CC; Germany	121 (48/73); 38.3 ± 14.3 yr. Cr = 59 (24/35); 38,2 ± 10 yr	CD = 46 UC = 16	Mucobuccal hyperplasia or oedema = 15, swelling of the gingiva = 17, ulcera = 5, Aphthae = 6, Candidiasis = 5, lichen planus = 3, leucoplachia=2, labial rhagades = 3	DMF-S (*p* 0.212 NS IBD = 54.1 ± 31.6. Cr = 46.5 ± 26.5 Dentine caries: (*p* = 0.033) IBD = 25 (40%); Cr = 13 (22%)	BOP (*p* = 0.958) NS IBD = 23.4 ± 20.1; Cr = 20.8 ± 13.5. PPD (*p* = 0.014) IBD = 2.22 ± 0.57; Cr = 2.29 ± 0.33. (*p* = 0.014) CAL > 4 mm: (*p* = 0.07) IBD = 50 (81%). Cr= 38. (64%) CAL > 5mm: (*p* = 0.07) IBD = 39 (63%) Cr = 27 (46%)	Corticosteroids, immunosuppressants, aminosalicylate, anti TNF, antibiotics	IBD nonsmokers = 34 (55%) IBD smokers = 25 IBD former smokers= 3 (5%); Cr nonsmokers = 29 (49%) Cr smokers = 24. Cr former smokers = 6 (10%)
**5**	Habashneh et al. [1]	CC; Jordania	260 (156/104); 39.4 ± 0.7 yr Cr = 100 (62/38)	CD = 59 (33/26) UC = 101 (61/40)	NN	NN	CD: PPD = 1.29 ± 0.47; CAL = 1.95 ± 0.98. % of sites with BOP = 10.84 ± 16.20 gingival recession = 0.53 ± 0.55 UC: PPD = 1.51 ± 0.47; CAL = 2.36 ± 1.13 % of sites with BOP = 10.20 ± 14.25 gingival recession = 0.86 ± 0.72 Cr; PPD = 1.25 ± 0.37, CAL = 1.70 ± 0.89 % of sites with BOP = 4.70 ± 8.30 gingival recession = 0.44 ± 0.60	NN	CD Nonsmoker = 23; Smoker = 31; ex-smoker = 5 UC nonsmoker = 55; smoker = 17; ex-smoker = 29 Cr nonsmoker = 44; smoker = 49; ex-smoker = 7
**6**	Harty et al. [12]	P; Ireland	80	CD = 49 (25/24); 11,95 yr UC = 22 ND = 9 oral CD = 20 (12/8) 11.4 yr non oral CD = 28	patients with oral CD compared to nonoral CD: oral symptoms = 14 (70%) (*p* = 0.01) mouth ulcers = 11 (55%) NS angular stomatitis = 5 (25%) NS cheek swelling=5 (25%) NS vomiting=2 (10%) NS	NN	nonspecific gingivitis = 8 (16.7%)	NN	NN
**7**	Jose et al. [5]	R/P; USA	1649 (893/756); 11.1 ± 4.15	CD = 1007. UC = 471. ND = 171	53 patients with aphthous stomatitis (13.7% of all EIM) before diagnoses	NN	NN	NN	NN
**8**	Katz et al. [14]	CC; Israel	96 (49/47); 38.5 ± 26.9 Cr:42 (22/20); 40 ± 20 yr	CD = 34 (20/14); 33 ± 16 yr UC = 20 (7/13); 44 ± 18 yr	Halitosis = 50% UC (*p* = 0.0008); 29% CD, (*p* = 0.026) dry mouth = 30% UC, (*p* = 0.04); 29% CD (*p* = 0.026) geographic tongue = 15% CD (*p* = 0.01), dysphagia = 15% UC, (*p* = 0.05); 9% CD NS nausea = 30% UC (*p* = 0.017), 50% CD (*p* = 0.001) vomiting = 15% UC, NS; 41% CD (*p* = 0.01) regurgitation = 45% UC,(*p* = 0.017); 21% CD, NS	NN	NN	NN	NN
**9**	Khouri et al. [4]	R; Australia	6 (5/1); 6.33 yr	CD = 4 M; 6.25 yr	Lip swelling = 6, granulomatous cheilitis = 6, cobblestoning mucosa = 2	NN	NN	NN	NN
**10**	Koutsochristou et al. [9]	CC; Greece	110 (50/60); 12.26 ± 5.22 yr Cr = 55 (25/30); 12.21 ± 3,96 yr	CD = 36 (18/18); UC = 19 (7/12); 13 of 55 patients had oral lesions (23%)	Aphthae = 8, aphthae with swelling of gums or ulcers or candidiasis = 5	DMFT IBD = 5.81 (*p* < 0.001) Cr = 2.04 dmft IBD = 2.95 (*p* < 0.001) Cr = 0.91	CPITN index: IBD: score 0 = 0 (0%) score 1 = 20 (36%) score 2 = 30 (54%) score 3 = 5 (9%) Cr: score 0 = 22 (40%) score 1 = 25 (45%) score 2 = 8 (14%) score 3 = 0 (0%)	aminosalicylates, corticosteroids, anti-TNF, or immuno-modulators	NN
**11**	Laranjeira et al. [15]	CC; Portugal	171 (85/86); 45.5 ± 16.9 yr. Cr = 58 (28/30); 47.4 ± 16.3 yr	CD = 65 (32/33); 41.1 ± 15.2 yr. UC = 48 (25/23); 49.2 ± 18.4 yr	Aphtous ulcers = 1 0 (8.80%), (*p* = 0.159 so NS); gingival swelling = 1 (0.90%), angular cheilitis = 1 (0.90%), halitosis (*p* = 0.038).	NN	NN	Corticosteroids,salicylate, immunosuppressants,	Non smokers Cr = 52 CD =52; UC = 42 Smokers Cr = 6 CD = 13; UC = 6
**12**	Mohan Kumar et al. [16]	CC; India	30 (16/14)	UC = 15 (8/7)	Aphtous ulcerations = 10 lichen planus = 3 dry mouth = 11 PSV = 1, coated tongue = 4 NS dysgeusia = 5, halitosis = 12	NN	Periodontal status NS: Periodontal index UC = 1. Cr = 1.4 gingival index UC = 1.2 Cr = 1.3 loss of attachment (mm) = UC 0.4; Cr = 0.2	Sulfapyridine; sulfasalazine	NN
**13**	Oviedo et al. [19]	T; Chile	30 (9/21); 40 yr	CD = 7 (2/5) UC = 23 (7/16)	11 patients (37%): Oral ulcerations = 1 Apthae = 4, angular ulcer = 1, macrocheilia of the lip, corrugated mucosa	NN	NN	NN	NN
**14**	Rikardsson et al. [17]	CC; Sweden	2346 (32.5%/67.5%); 49.6 ± 20, 60 Cr = 748 (33%/67%); 49.5 yr ± 13.8 yr	CD = 1598 (32%/68%); 49.7 ± 15.3 yr	Oral ulcers = 32% (*p* < 0.001), halitosis = 23% (*p* < 0-001) mouth dryness = 38% (*p* < 0.001) toothaches = 21% (*p* < 0.001) mucosal lesions = 31% (*p* < 0.001)	Carious lesions = 41% (*p* < 0.001)	bleeding from gingiva = 41% (*p* < 0.001) periodontitis = 7% (*p* < 0.028)	NN	Current smokers CD = 23% Cr = 19% (*p* < 0.018) former smokers CD = 19% Cr = 15% (*p* < 0.070)
**15**	Szymanska et al. [8]	CC; Sweeden	225 (102/123); 47.1 ± 24.08 yr Cr = 75 (29/46); 48.6 ± 13.4 yr	CD with RS = 71 (33/38); 50.7 ± 13.9 yr CD without RS = 79 (40/39); 42 ± 14.4 yr	Dry mouth (*p* = 0.001): 11% in Cr,29% in CD with RS and 38% in CD with NRS Bad breath (*p =* 0.008):12% in Cr, 21% in CD with RS and 33% in CD with NRS Ulcers (NS): 23% in Cr, 23% in RS and 27% in CD with NRS	Cr = 13.1 CD with RS = 15,5. CD with NRS = 11.2	NN	NN	Cr = 5 CD with RS = 17 CD with NRS = 15 Former smokers Cr = 33 CD with RS = 25 CD with NRS = 30
**16**	Zervou et al. [18]	CC; Greece	74; 41,5 ± 20 yr. Cr = 47;43 ± 12 yr	CD = 15 UC = 15	Ulcers = 3 (2 CD,13% (*p =* 0.011); 1 UC,7%, (*p* = 0.07)) cobblestoning = 3 CD,20%, (*p* = 0.002) polypoids tags = 3 CD,20%, (*p* = 0.002) lip swelling = 4 (3 CD,20%, (*p* = 0.002); 1 UC, 7%, (*p* = 0.07)) buccal swelling = 1 CD,7%, (*p* = 0.07) aphthous ulcers = 1 UC,7%, (*p* = 0.07) angular cheilitis = 9 (5 CD, 33% (*p* = 0.000); 4 UC,27%, (*p* < 0.0001)) Hairy tongue = 3 (2 CD, 13%, (*p* = 0.011); 1 UC (*p* = 0.07)) buccal trauma = 9 (6 CD, 40%, (*p* = 0.000); 3 UC, 20% (*p* < 0.00019))	NN	Periodontitis=2 CD 13%, (*p* = 0.011) Gingivitis = 4 (3 CD, 20%, (*p* = 0.002); 1 UC, 7%, (*p* = 0.07)) Gingival bleeding = 4 CD 27% (*p* < 0.0001)	Mesalazine, aziathioprine	NN
**17**	Zippi et al. [3]	R; Italy	811 (437/374); 32.5 ± 18,9 EIMs = 329 (155/174) (210 UC;119 CD)	CD = 216 (131/85); 31,9 ± 13,1 yr. UC = 595 (306/289); 33,1 ± 13,7 yr	6 cases of aphtous stomatitis, 3 CD e 3 UC (1.4% CD e 0.5% UC)	NN	NN	NN	CD Smoker = 98. Non-smoker = 101 Ex smokers = 17. UC Smokers = 140. Ex smokers = 131. Non smokers = 324

Legend: DMFT = decayed, missing, filled teeth index in the permanent dentition; dmft = decayed, missing, filled teeth index in the primary dentition; EIMs = extra intestinal manifestations; NN = unknown; NS = not statistical; *p* = p value; P = prospective study; CC = case control; T = cross sectional study; NRS = non resective surgery ; R = retrospective study; RS = resective surgery; ND = indeterminate colitis.

**Table 6:** Specific and nonspecific manifestations of IBD.

**Specific manifestations**	**Oral manifestations**	**Crohn’s disease**	**Ulcerative colitis**
	Cobblestoning the mucosa	X	
	Granulomatous cheilitis	X	
	Mucosal tags	X	
	Pyostomatitis vegetans		X
**Unspecific manifestations**	Deep oral fissuring	X	
	Cheilitis angularis	X	X
	Dental caries	X	X
	Mucogingivitis	X	X
	Periodontitis	X	X
	Lichen planus	X	X
	Dysphagia	X	X
	Dry mouth	X	X
	Halitosis	X	X
	Taste changes	X	X
	Aphthous ulcerations	X	X

Legend: X = presence of the manifestation.
